# Visualizing endoderm cell populations and their dynamics in the mouse embryo with a *Hex-tdTomato* reporter

**DOI:** 10.1242/bio.024638

**Published:** 2017-03-13

**Authors:** Tao Wu, Anna-Katerina Hadjantonakis, Sonja Nowotschin

**Affiliations:** Developmental Biology Program, Sloan Kettering Institute, Memorial Sloan Kettering Cancer Center, 1275 York Avenue, New York, NY 10065, USA

**Keywords:** Hex, AVE, Visceral endoderm, Definitive endoderm, Live imaging, Gastrulation, Red fluorescent protein

## Abstract

Live imaging is the requisite tool for studying cell behaviors driving embryonic development and tissue formation. Genetically encoded reporters expressed under cell type-specific *cis*-regulatory elements that drive fluorescent protein expression at sufficient levels for visualization in living specimens have become indispensable for these studies. Increasingly dual-color (red-green) imaging is used for studying the coordinate behaviors of two cell populations of interest, identifying and characterizing subsets within broader cell populations or subcellular features. Many reporters have been generated using green fluorescent protein (GFP) due to its brightness and developmental neutrality. To compliment the large cohort of available GFP reporters that label cellular populations in early mouse embryos, we have generated a red fluorescent protein (RFP)-based transgenic reporter using the red fluorescent tdTomato protein driven by *cis*-regulatory elements from the mouse *Hex* locus. The *Hex-tdTomato* reporter predominantly labels endodermal cells. It is a bright RFP-based reporter of the distal visceral endoderm (DVE)/anterior visceral endoderm (AVE), a migratory population within the early post-implantation embryo. It also labels cells of the definitive endoderm (DE), which emerges at gastrulation. Dual-color visualization of these different early endodermal populations will provide a detailed understanding of the cellular behaviors driving key morphogenetic events involving the endoderm.

## Abstract (precis)

**Summary:** A red fluorescent reporter under the regulatory control of the mouse *Hex* gene permits identification of different endodermal populations and visualization of dynamic cellular behaviors driving endoderm specification and morphogenesis.

## INTRODUCTION

Fluorescent reporters are an essential tool for live imaging, quantitation and understanding of complex cellular dynamics during development and tissue morphogenesis. The green fluorescent protein (GFP) and its yellow fluorescent (YFP) variants have been the reporters of choice due to their brightness and their photostability compared to other spectrally distinct fluorescent proteins (Abe and Fujimori, 2013; Nowotschin et al., 2009; Shaner et al., 2005). Though single color imaging has yielded many key insights, it has become increasingly clear that visualization of additional cellular populations or multiple subcellular features within individual cells through dual- or multi-spectral imaging is the way forward in the study of complex and coordinated *in vivo* cellular behaviors. Coordinate visualization, afforded by the use of one reporter, also provides a tool for the isolation of distinct subsets of cells existing within larger more complex populations for studies such as transcriptomic analyses.

To complement the large cohort of existing GFP-based reporters, red fluorescent proteins (RFP) are the most suitable for dual-color imaging, since they emit at a longer wavelength part of the light spectrum. RFPs are ideal in combination with GFPs since they can easily be spectrally separated on most fluorescent imaging systems. In addition, RFPs are less phototoxic than GFPs, and given their longer wavelength excitation/emission facilitate a deeper penetrance into tissue. Amongst a number of characterized RFPs, tandem-dimer(td)Tomato is bright and photostable when visualized with a variety of optical imaging modalities (Shaner et al., 2004, 2005). Given its exceptional brightness, which has been reported as being up to eight times brighter than GFP, tdTomato is ideal for *in vivo* studies and has been successfully applied to label diverse cell types in the mouse, and has been incorporated into two popular recombinase-activated reporter alleles, *R26^(ACTB-tdTomato,-EGFP)Luo^* (Muzumdar et al., 2007) and *R26^(CAG-tdTomato)Hze^* (Madisen et al., 2010). For these reasons we chose to generate a tdTomato-based reporter that would label various endodermal cell types, including the primitive and definitive endoderm lineages of early mouse embryos (Chazaud and Yamanaka, 2016; Viotti et al., 2014a). We reasoned that a tdTomato-based reporter, when combined with existing GFP-based reporters, would facilitate dual-color imaging, the next step in live imaging based experimental approaches.

The first endodermal cell type to arise in the mouse embryo is the primitive endoderm (PrE) which is specified at embryonic day (E)3.5 in the blastocyst stage embryo (Chazaud and Yamanaka, 2016). The PrE will predominantly give rise to the extra-embryonic endodermal tissue derivatives, including the extra-embryonic visceral endoderm (exVE), contributing later on to the endoderm layer of the visceral and parietal yolk sacs (Nowotschin and Hadjantonakis, 2010), as well as the embryonic visceral endoderm (emVE) overlying the epiblast, that contributes cells to the embryonic gut endoderm (Kwon et al., 2008). At E5.0-E5.5 a subset of cells at the distal tip of the embryo resident within the emVE layer acquire a columnar morphology and differentiate into the distal visceral endoderm (DVE) (Arnold and Robertson, 2009; Rivera-Perez and Hadjantonakis, 2014). A hallmark of these cells is the expression of specific marker genes including *Hex* [haematopoietically expressed homeobox (Thomas et al., 1998)], *Lefty1* [left-right determination factor 1 (Meno et al., 1998; Yashiro et al., 2000)], and *Cer1* [Cerberus-like 1 (Belo et al., 1997)]. By E5.5, a second group of cells, the anterior visceral endoderm (AVE), is specified in close proximity to the DVE at the distal tip of the embryo expressing the same marker genes (Takaoka and Hamada, 2014). Both, DVE and AVE are marked by *Hex* gene expression as they coordinately migrate away from the distal tip of the embryo (Takaoka et al., 2011). This unilateral collective cell migration defines the future anterior side of the embryo. DVE/AVE migration comes to a halt when the population reaches the extra-embryonic-embryonic boundary. Once they reach this boundary, DVE cells reorient their direction of migration as the population bifurcates to the left and right sides of the embryo with each branch continuing its movement along a lateral-distal trajectory (Srinivas et al., 2004; Takaoka et al., 2011).

Studies of the cellular behaviors of AVE/DVE cells have relied on live imaging of genetically encoded fluorescent protein reporters that are controlled by AVE/DVE-specific genes. To date, three transgenic reporters have been published: *Hex-GFP*, *Cerl-GFP* and *Lefty1-mVenus* (Mesnard et al., 2004; Rodriguez et al., 2001; Takaoka et al., 2011). In this study we used the *Hex* regulatory elements to generate a reporter that is spectrally distinct, and thus can be combined with existing reporters for dual-color visualization. We used the characterized *cis*-regulatory elements of the *Hex* gene (Rodriguez et al., 2001). Downstream targets of *Hex* include *Cyclin D1*, *Tle4* and *Nodal*, suggesting roles for *Hex* in controlling cell cycle and signaling pathways (Soufi and Jayaraman, 2008; Topisirovic et al., 2003; Zamparini et al., 2006). In mouse embryos, *Hex* is expressed in cells of the PrE lineage, as well as the definitive endoderm (DE), the second endodermal population to be established in the embryo (Viotti et al., 2014a). *Hex* is also expressed in endothelial cell precursors where it is essential for hematopoietic and vascular differentiation (Martinez Barbera et al., 2000). Consistent with its pattern of expression, *Hex* null mutants are embryonic lethal exhibiting defects in the formation of the liver, thyroid, ventral pancreas buds and the forebrain (Martinez Barbera et al., 2000). The functions of *Hex* appear to be evolutionary conserved with its homologs in the chick (*Gallus gallus*) and frog (*Xenopus laevis*) expressed in tissues analogous with the mouse DVE/AVE and DE, where comparable morphogenetic movements of these cellular populations have been described (Chapman et al., 2002; Jones et al., 1999; Smithers and Jones, 2002).

The mouse *Hex* promoter contains multiple *cis*-regulatory elements, which coordinately direct *Hex* gene expression in various tissues at different developmental stages. The 4.2 kb region upstream of the transcriptional start site (TSS) in the *Hex* gene is necessary for expression in endothelial cell precursors, liver and thyroid. While a 633 bp fragment located within the third intron is crucial for expression in DVE/AVE and DE cell populations. The construct used to make the widely employed *Hex-GFP* transgenic mouse line contains all these regulatory regions, and appears to faithfully recapitulate endogenous *Hex* gene expression across different developmental stages (Rodriguez et al., 2001). This *Hex-GFP* mouse has been instrumental for studying the behaviors and functions of AVE/DVE cells (Migeotte et al., 2010; Nowotschin et al., 2013; Rakeman and Anderson, 2006; Trichas et al., 2011). Although this reporter has been pivotal in providing insight into the organization and dynamic behaviors of DVE/AVE cells within early mouse embryos, its use for dual-labeling experiments has been limited due to its sole availability as a green fluorescent variant. Consequently, understanding the coordination between AVE/DVE cells and their neighbors within the emVE and the epiblast and its derivatives remain unexplored. For this reason, we sought to generate a spectrally-distinct RFP-based reporter employing the well characterized and widely used *Hex* cis-regulatory elements. The transgenic *Hex-tdTomato* mouse line we have generated enables dual-color live imaging of distinct endoderm cell populations and their respective cellular behaviors. Furthermore, this mouse line when used in combination with other spectrally-distinct cell type-specific reporters should permit the identification and isolation of different cell populations comprising the visceral endoderm (VE) for transcriptomic analyses.

## RESULTS AND DISCUSSION

### Generation and validation of a *Hex-tdTomato* reporter construct

**Fig. 1.**
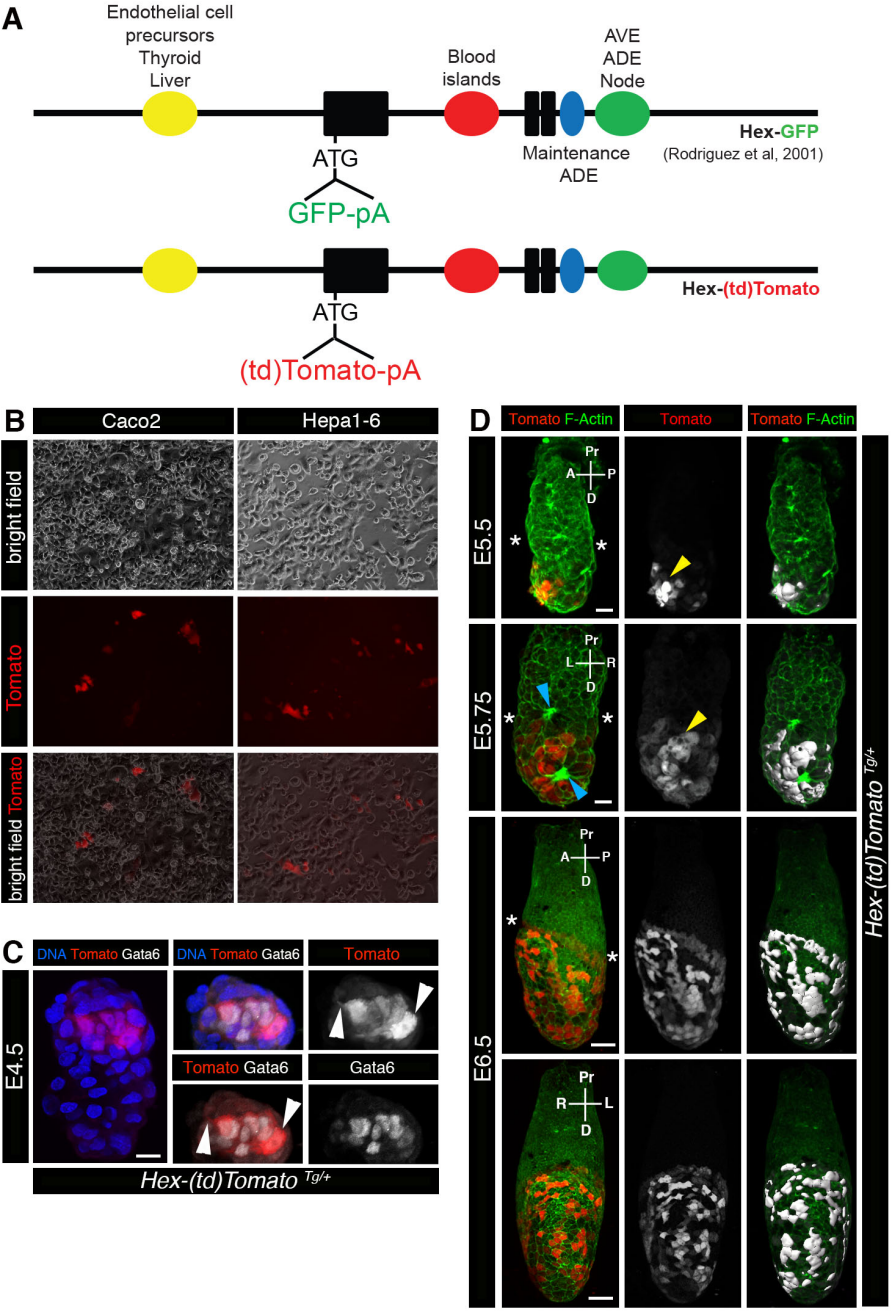
***Hex-tdTomato* construct design and validation in cells and early mouse embryo.** (A) Schematic representations of the previously published *Hex-GFP* construct (Rodriguez et al., 2001) and *Hex-tdTomato* construct depicting the *cis*-regulatory elements and the inserted fluorescent protein cassette used to generate the *Hex-tdTomato* transgenic mouse line. (B) Validation of the expression of the *Hex-tdTomato* construct in two endoderm derived cell lines, Caco2 and Hepa 1-6. Tomato expression can be seen in both cell lines. (C) Expression of Tomato can be detected in Gata6-positive primitive endoderm cells at the late blastocyst stage (E4.5). Note, cytoplasmic Tomato expression highlights primitive endoderm cells extending filopodia (white arrowheads). Panels show 3D renderings of laser confocal images of a whole mount view immunostained for Tomato, Gata6 and Hoechst (DNA), and zoomed-in views of the ICM depicting, Hoechst (DNA), Tomato and Gata6 3-channel merge, Tomato and Gata6 expression 2-channel merge and Tomato and Gata6 single channel images. (D) Expression of Tomato at early post-implantation stages E5.5 and E6.5. Right panels show 3D renderings of laser confocal images of Tomato reporter and F-Actin expression, center panels show Tomato expression only, left panels show 3D surface rendering of Tomato expression counterstained with F-Actin. Yellow arrowheads point to leading cells expressing elevated levels of fluorescence. Blue arrowheads point to rosette-like cell arrangements in the embryonic VE. ADE, anterior definitive endoderm; A, anterior; AVE, anterior visceral endoderm; D, distal; P, posterior; Pr, proximal. Asterisk marks the border of extra-embryonic and embryonic visceral endoderm. Scale bars: (C) 10 µm; (D) 20 µm (E5.5); 20 µm (E5.75); 50 µm (E6.5).

An 8 kb enhancer fragment containing the regions 4.2 kb upstream and 3.8 kb downstream of the transcriptional start site ATG of the mouse *Hex* gene has been shown to be sufficient to drive transgene expression in the endothelial precursors of the thyroid and the liver, the blood islands as well as the AVE, anterior DE (ADE) and node (Rodriguez et al., 2001). A *Hex-tdTomato* construct was generated using this 8 kb element to drive expression of the tdTomato red fluorescent protein (Shaner et al., 2004), in a design that was comparable to the well characterized and widely used *Hex-GFP* reporter (Rodriguez et al., 2001) (Fig. 1A). The endoderm specificity of the *Hex-tdTomato* construct was validated by transfection into Caco2 (human colon) and Hepa1-6 (mouse liver) cell lines. Both cell lines expressed tdTomato upon transfection (Fig. 1B). Having confirmed its specificity and readily detectable expression in cell lines of endodermal origin, the *Hex-tdTomato* construct was used to generate transgenic mice by pronuclear injection of zygotes. Five founder transgenic animals were recovered. These were screened for correct and readily detectable Tomato expression in embryos. Two of the founder lines (#7 and #41) produced faithful *Hex-tdTomato* expression at all stages of embryos analyzed. Images presented in this manuscript are of hemizygous embryos of the line established from founder #7.

### *Hex-tdTomato* reporter marks the PrE lineage of the blastocyst

Transgenic mice (males or females) were crossed to wild-type animals and hemizygous embryos were analyzed for expression of tdTomato at various stages of endoderm development. Tomato-positive cells were first detected in late blastocyst stage (>100-cell, E4.5) embryos. The location of the tdTomato-positive cells, combined with their expression of Gata6, identified them as PrE (Fig. 1C). This observation is in agreement with previously reported PrE-specific *Hex* gene expression (Thomas et al., 1998), as well as *Hex* reporter expression observed in cells with a PrE identity within embryonic stem cell cultures (Canham et al., 2010; Morrison et al., 2008). To date, the *Hex-tdTomato* line is the first cytoplasmic RFP-based reporter that marks the PrE lineage of the mouse blastocyst. As a cytoplasmic reporter it revealed filopodial extensions present on some PrE cells (white arrowheads in Fig. 1C). This suggested that these PrE cells might be actively modulating their shape prior to their apical-basal polarization and formation of the PrE epithelium in the (late) implanting blastocyst (Chazaud and Yamanaka, 2016; Gerbe et al., 2008).

A *Hex-GFP* reporter has been extensively used as a tool to study DVE formation and AVE migration at peri- and early post-implantation stages of mouse embryo development (Rodriguez et al., 2001; Srinivas et al., 2004). In agreement with previous studies, Tomato-positive cells could be detected in a small patch situated at the distal tip of the embryo, presumably representing the DVE, at early E5.5. As with the *Hex-GFP* reporter, heterogeneity in Tomato expression was noted in DVE/AVE cells, with some cells expressing lower levels of Tomato than others (Fig. 1D, top row). This heterogeneity in the expression of genes associated with the DVE/AVE has been previously reported, and has also been observed in the expression of genes such as *Cerl* and *Hex* (Srinivas et al., 2004). However such a heterogeneity could also be attributed to the site of transgene integration, and a position-effect associated with the transgene, as noted in our comparison of *Hex-tdTomato* and *Hex-GFP* expression, which revealed some minor differences. Even so, cells at the anterior leading edge of the DVE/AVE population consistently expressed elevated levels of fluorescence than trailing cells (yellow arrowheads in Fig. 1D). This is consistent with observations made with a *Cerl-GFP* reporter (Morris et al., 2012). At E5.75 AVE cells had started migrating towards the future anterior side of the embryo (Fig. 1D, second rows). During this phase, additional numbers of cells within the AVE started expressing the *Hex-tdTomato* reporter (second row in Fig. 1D). As previously described (Trichas et al., 2012), rosette-like cell arrangements could be seen at the anterior side of the embryo at this time (blue arrowheads in Fig. 1D). At E6.5 Tomato-positive cells could be detected at the extra-embryonic/embryonic border consistent with their anterior migration having been halted as the population bifurcated, and extended laterally (to the left and right side of the embryo). Additional patches of Tomato-positive VE cells located on the lateral side could be seen at this stage (Fig. 1D, bottom two panels).

### Expression of *Hex-tdTomato* in axial mesendoderm and DE cells

**Fig. 2.**
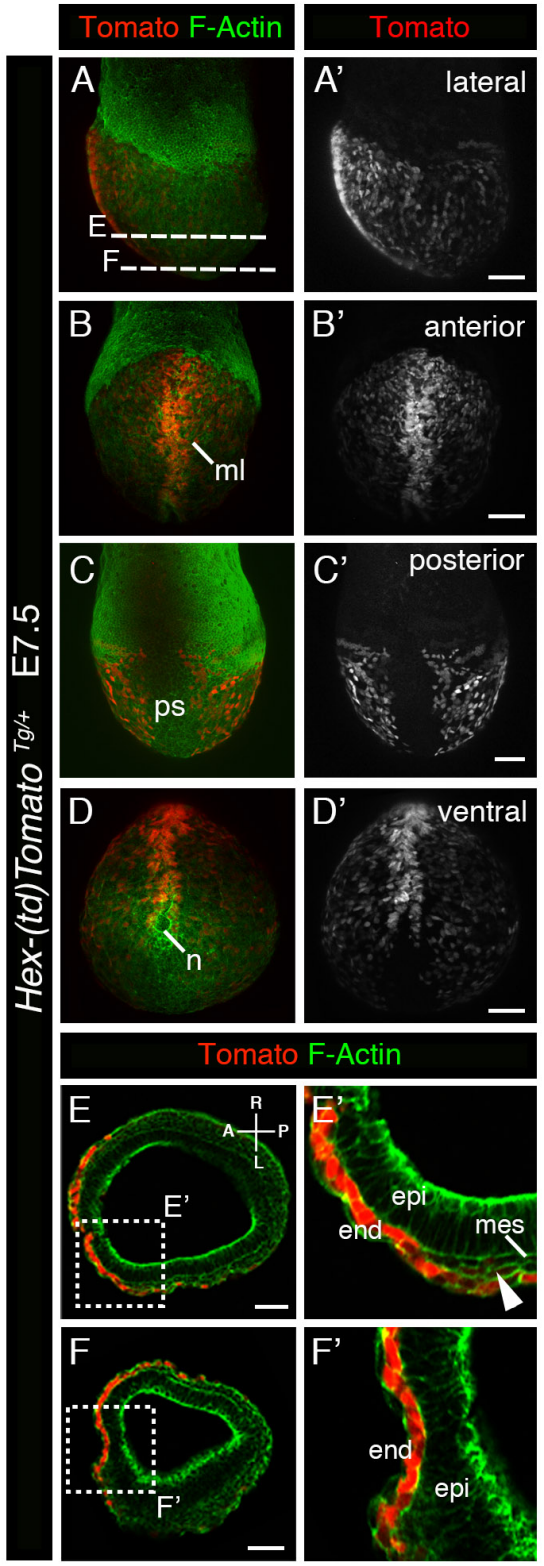
***Hex-tdTomato* is expressed in cells of the embryonic visceral endoderm and definitive endoderm at E7.5.** (A-D′) Lateral (A), anterior (B), posterior (C) and ventral (D) whole mount views of an E7.5 *Hex-tdTomato* embryo. Panels on the left (A-D) show 3D renderings of laser confocal images of Tomato reporter and F-Actin expression and on the right (A′-D′) Tomato expression only. (E,F) Transverse sections through the embryonic part of an E7.5 *Hex-tdTomato* embryo showing expression of Tomato specific to endodermal cells. (E′,F′) High magnification images of squares in E and F. Arrowhead points to expression in definitive endoderm cells. end, endoderm; epi, epiblast; mes, mesoderm; ml, midline; n, node; ps, primitive streak. Scale bars: (A-D′) 100 µm; (E-F′) 50 µm.

At mid-gastrulation (E7.5) strong expression of the *Hex-tdTomato* reporter was observed in cells situated at the midline, representing the axial mesendoderm (Fig. 2B), as shown previously by *Hex-GFP* expression (Rodriguez et al., 2001). Cells surrounding the node were also Tomato-positive, however, the cells comprising the node itself were devoid of Tomato expression (Fig. 2D). VE cells on the lateral side of the embryo continued to express the reporter though at reduced levels compared to earlier stages (Fig. 2A). By contrast, no expression was detected in VE cells overlying the primitive streak (Fig. 2C). Transverse sections of embryos at E7.5 revealed reduced levels of Tomato expression in DE cells (Fig. 2E′, white arrowhead), in addition to the more robust expression in the midline and VE (Fig. 2E,F).

**Fig. 3.**
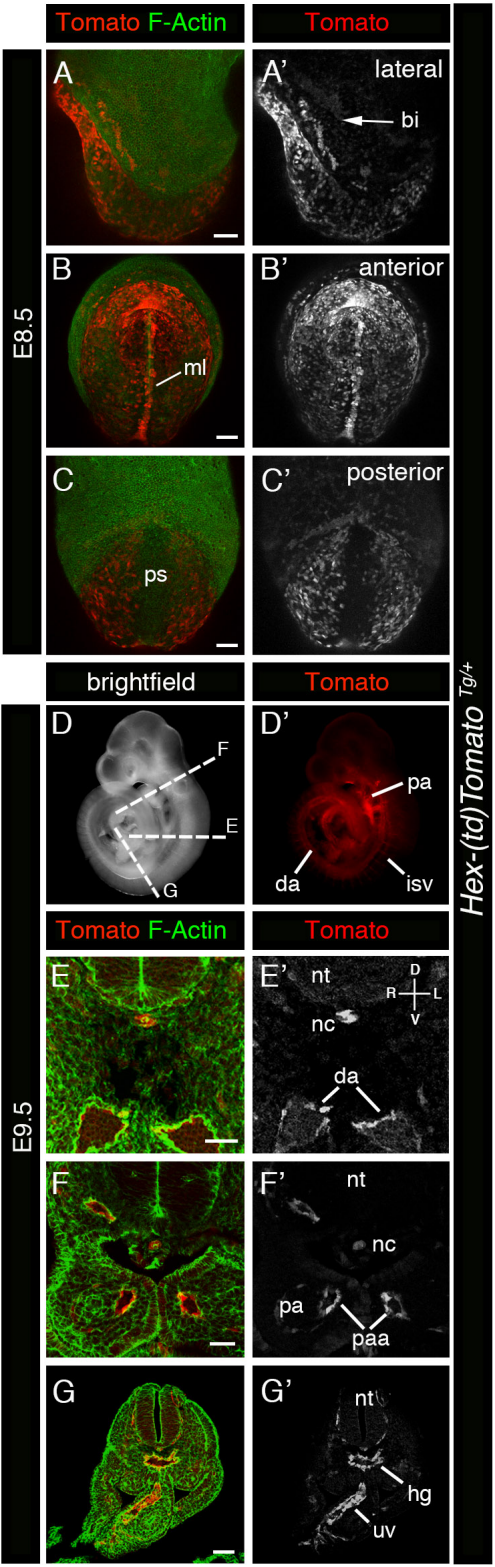
**Expression of *Hex-tdTomato* at E8.5 and E9.5.** (A-C′) Lateral (A), anterior (B) and posterior (C) whole mount views of an E7.5 embryo. (A-C) 3D renderings of laser scanning confocal images of tdTomato reporter co-stained with F-Actin. (A′-C′) Tomato expression only. (D,D′) Whole mount bright field and red fluorescent views of an E9.5 *Hex-tdTomato* embryo. (E-G′) Laser confocal images of transverse sections through an E9.5 *Hex-tdTomato* embryo showing Tomato expression co-stained with F-Actin (E-G) and (E′-G′) Tomato expression only. bi, blood islands; D, dorsal; da, dorsal aorta; L, left; hg, hindgut; isv, intersomitic vessels; nc, notochord; nt, neural tube; pa, pharyngeal arch; paa, pharyngeal arch artery; ps, primitive streak; R, right; uv, umbilical vein; V, ventral. Scale bars: (A-C) 100 µm; (E-G) 50 µm.

At early somite stages (E8.5) the majority of gut endoderm cells were Tomato-positive (Fig. 3A,B,C). The axial midline, representing the future notochord, retained expression of the reporter at elevated levels (Fig. 3B). Lower levels of expression could also be detected at this stage in the forming blood islands in the extra-embryonic yolk sac region of the embryo (Fig. 3A, arrow). By E9.5, Tomato expression was observed in endothelial cells within the dorsal aorta, as well as the pharyngeal arch arteries, intersomitic vessels, and the umbilical vessels. Tomato expression was also detected in the notochord, foregut diverticulum and hindgut (Fig. 3D′-G′), in concordance with previous observations (Rodriguez et al., 2001; Thomas et al., 1998).

### Correlation between *Hex-tdTomato* and *Hex-GFP* reporter expression

**Fig. 4.**
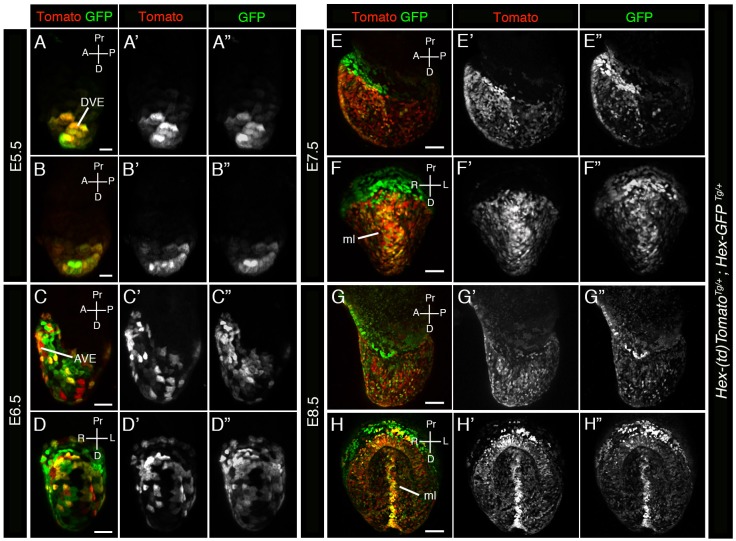
**Correlation in *Hex-tdTomato* and *Hex-GFP* reporter expression.** (A-H′′) 3D renderings of laser scanning confocal images of whole mount views of E5.5 (A,B′′), E6.5 (C,D′′), E7.5 (E,F′′) and E8.5 (G,H′′) *Hex-tdTomato; Hex-GFP* embryos expressing Tomato and GFP. (A-H) Panels show Tomato and GFP expression. (A′-H′) Panels show Tomato expression. (A″-H″) Panels show GFP expression. A, anterior; AVE, anterior visceral endoderm; D, distal; DVE, distal visceral endoderm; ml, midline; P, posterior; Pr, proximal. Scale bars: (A,B) 20 µm; (C,D) 50 µm; (E-H) 100 µm.

To validate the *Hex-tdTomato^TG/+^* reporter as a faithful reporter of *Hex* gene expression, we sought to establish whether it exhibited comparable expression to the well characterized and widely used *Hex-GFP* reporter (Rodriguez et al., 2001). To do so, we generated *Hex-tdTomato*; *Hex-GFP* double transgenic reporter animals. We analyzed double hemizygous transgenic embryos at early post-implantation stages and examined the expression of both GFP and Tomato and determined the existence and extent of co-localization (Fig. 4). At E5.5 the two reporters exhibited an indistinguishable pattern of transgene expression, however some cells exhibited higher levels of Tomato fluorescence while others exhibited higher levels of GFP fluorescence (Fig. 4A,B), suggesting some aspect of *Hex* expression heterogeneity within the population. At later stages, E6.5 through E8.5, expression of the two reporters continued to be mostly overlapping, with a few exceptions. Some reporter-expressing cells again exhibited higher GFP than Tomato expression or vice versa, while others exhibited comparable expression. Some cells were only Tomato-positive whereas others only GFP-positive. At E7.5, as well as at E8.5, strong expression of the *Hex-GFP* reporter could be detected in the anterior extra-embryonic region, at the embryonic/extra-embryonic border, whereas Tomato expression was barely detectable at E7.5 (Fig. 4E,F); however, by E8.5 some of these cells had also started to express Tomato. These cells have been described previously (Srinivas et al., 2004) and have been hypothesized to originate from emVE that has been displaced to extra-embryonic regions during gut endoderm formation. However, subsequent studies have shown that the VE is dispersed rather than displaced (Kwon et al., 2008; Viotti et al., 2014b), so if such a displacement occurs it might only involve emVE cells situated close to the embryonic/extra-embryonic border. Alternatively, it has been suggested that since the extra-embryonic ectoderm has been replaced by the forming yolk sac at this time, and since the latter may no longer produce a repellant signal for them, AVE cells might cross the embryonic/extra-embryonic border, or alternatively AVE cells may themselves have changed as shown by the downregulation of *Hex* gene expression (Srinivas et al., 2004). Since we could not detect a strong signal in these cells with our *Hex-tdTomato* reporter, downregulation of the *Hex* gene and a change in the properties of AVE cells seems to be a likely explanation. Expression of *Hex-GFP* in these cells may result from the longer perdurance of the GFP protein compared to tdTomato. However, we can not rule out the possibility that differences in expression of the two transgenes due to position effect variegation or due to heterogeneity in transgene expression.

### *Hex-tdTomato* labels a subset of visceral endoderm cells

**Fig. 5.**
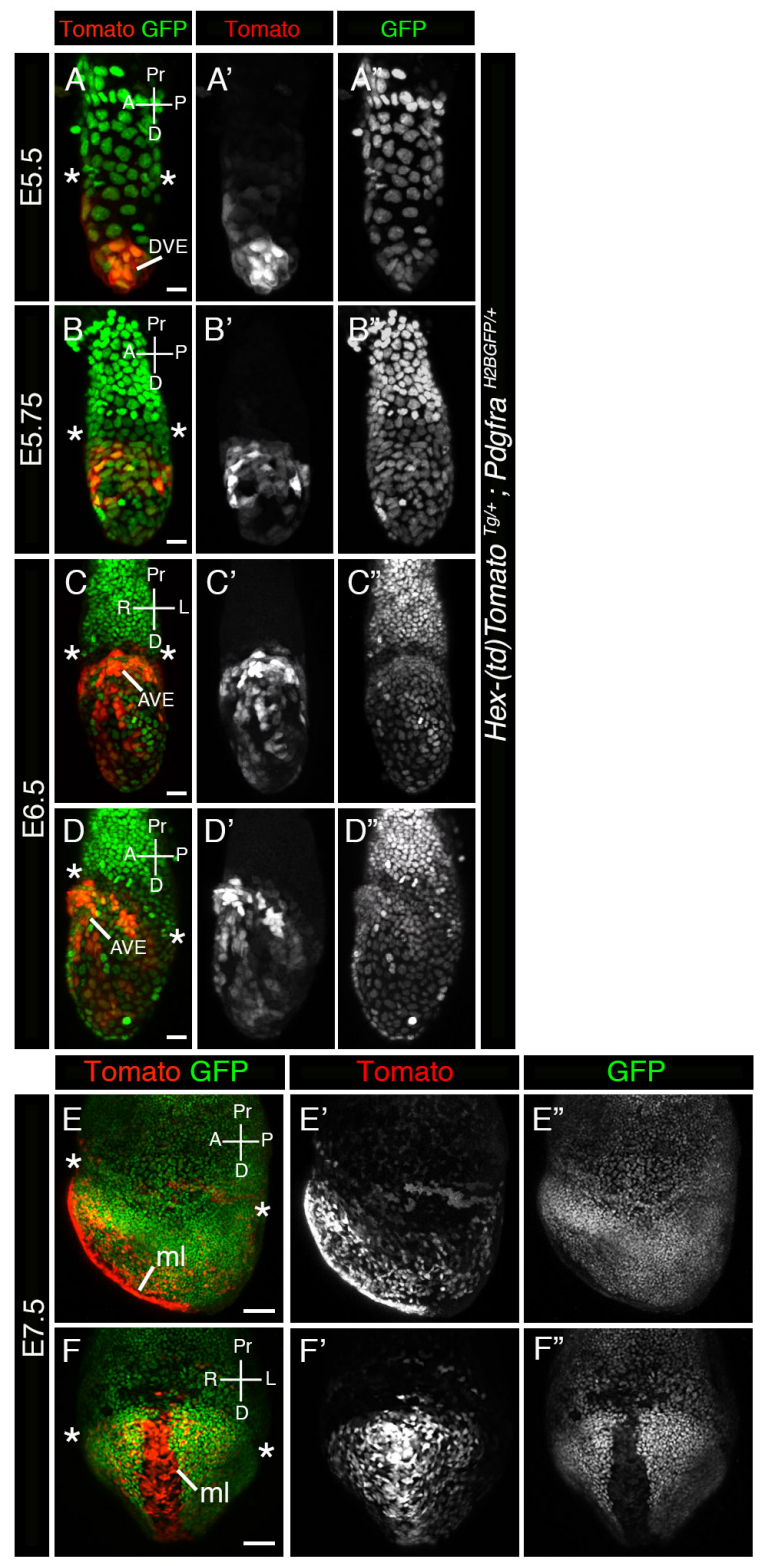
***Hex-tdTomato* reporter labels a subpopulation of *Pdgfra^H2BGFP^*-positive VE cells, as well as nascent DE and midline cells.** (A-F″) 3D renderings of laser scanning confocal images of whole mount views of E5.5 (A-A″), E5.75 (B-B″), E6.5 (C-D″) and E7.5 (E-F″) *Hex-tdTomato; Pdgfra^H2BGFP^* embryos expressing Tomato and GFP. (A′-F′) Tomato expression and (A″-F″) GFP expression. Asterisks mark the border of extra-embryonic and embryonic visceral endoderm. A, anterior; AVE, anterior visceral endoderm; D, distal; DVE, distal visceral endoderm; ml, midline; P, posterior; Pr, proximal. Scale bars: (A,B) 20 µm; (C,D) 50 µm; (E,F) 100 µm.

Live-imaging two spectrally-distinct reporters that label discrete subcellular compartments within the same cell population, or which are expressed in two distinct populations, will enable the study of coordinate cell behaviors, and identify, quantify and isolate subsets of cell populations. To this end, we crossed the *Hex-tdTomato* reporter with the *Pdgfra^H2BGFP/+^* line, a nuclear-localized H2BGFP reporter knock-in into the *Pdgfra* locus (Hamilton et al., 2003). *Pdgfra* is a marker of PrE cells in the blastocyst (Plusa et al., 2008), and a marker of embryonic and extra-embryonic VE cells at peri- and early post-implantation stages (Artus et al., 2010). At early gastrulation stages *Pdgfra^H2BGFP/+^* labels the extra-embryonic VE and nascent mesoderm as it emerges at gastrulation (Viotti et al., 2014b). Imaging of a series of early post-implantation stage *Hex-tdTomato*; *Pdgfra^H2BGFP/+^* double transgenic embryos revealed that the *Hex-tdTomato* reporter was expressed in a subset of *Pdgfra*-positive cells at E5.5 and E6.5 (Fig. 5A-D), in DVE/AVE cells, and during AVE cell migration. At E7.5 GFP was expressed in cells comprising the wings of mesoderm, which did not express Tomato. However, tdTomato expression was detected in DE cells which were GFP-negative as they traveled along the wings of mesoderm before intercalating into the overlying VE epithelium. Similarly, axial mesendoderm cells were positive for Tomato, but not GFP (Fig. 5E,F).

**Fig. 6.**
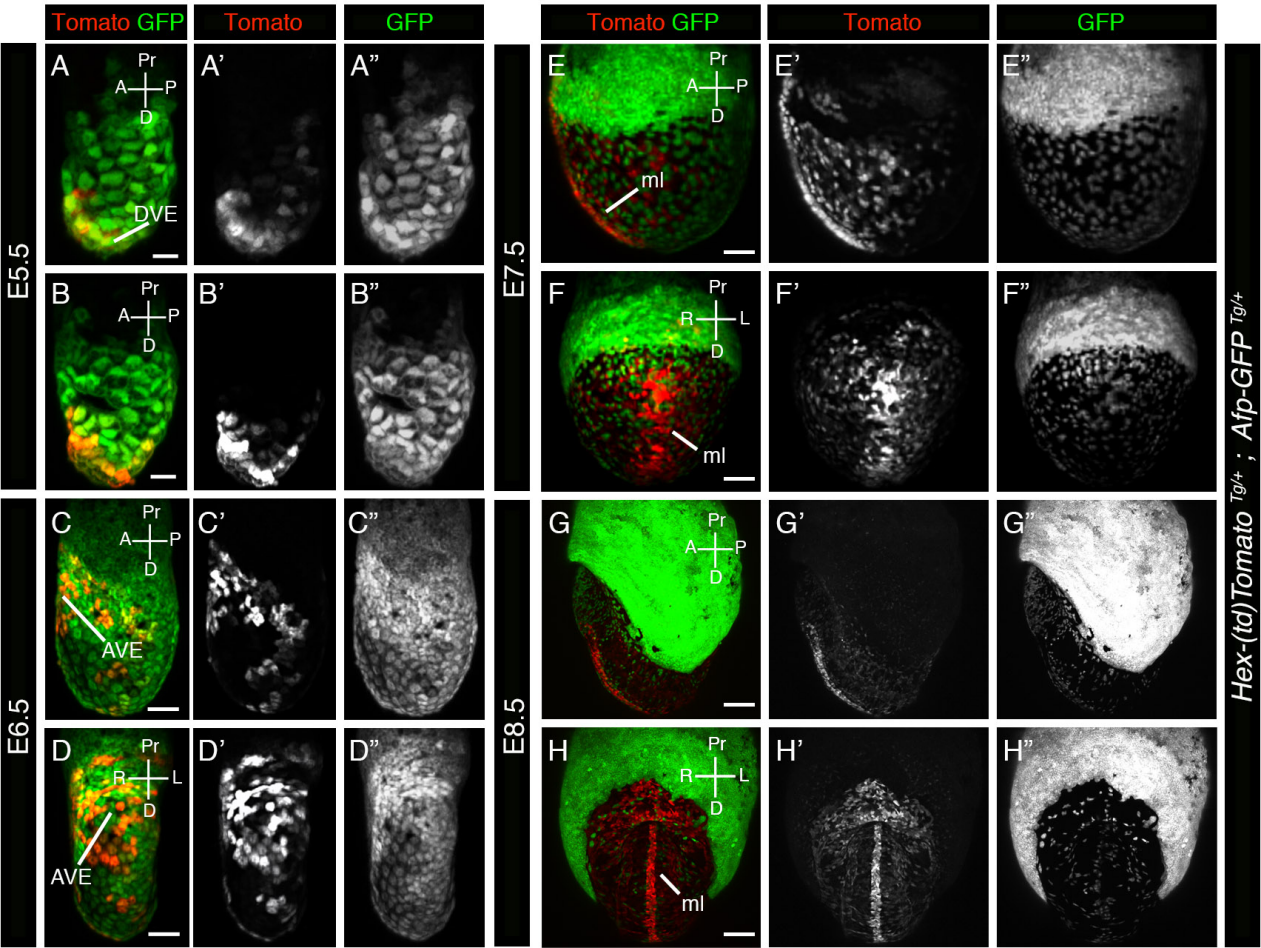
***Hex-tdTomato* marks a subset of AFP-GFP-positive VE cells.** 3D renderings of whole mount views of (A-B′′) E5.5, (C-D′′) E6.5, (E-F′′) E7.5 and (G-H′′) E8.5 *Hex-tdTomato; AFP-GFP* embryos. Panels show expression of (A-H) Tomato and GFP, (A′-H′) Tomato and (A″-H″) GFP. A, anterior; AVE, anterior visceral endoderm; D, distal; DVE, distal visceral endoderm; L, left; ml, midline; P, posterior; Pr, proximal; R, right. Scale bars: (A,B) 20 µm; (C,D) 50 µm; (E-H) 100 µm.

We also crossed the *Hex-tdTomato* reporter with our *Afp-GFP* line, in which a cytoplasmic GFP is expressed under the *cis*-regulatory elements of the *Alpha fetoprotein* (*Afp*) locus driving transgene expression throughout the VE, and later, DE derivatives (Kwon et al., 2006); alpha fetoprotein (Afp) is a serum protein secreted by VE cells (Belayew and Tilghman, 1982). When imaging the *Hex-tdTomato*; *Afp-GFP* double transgenic dual reporter expressing embryos, we noted overlapping expression in a subset of VE cells at E5.5 and E6.5. Co-expression of Tomato and GFP was only detected in the DVE and AVE cells at E5.5 (Fig. 6A,B) and in AVE and embryonic/extra-embryonic border cells at E6.5 (Fig. 6C,D). Once gastrulation had started, GFP and Tomato were generally mutually exclusive with a small population of dual-labeled cells. The AFP reporter was localized to exVE and emVE cells. This would suggest that AVE cells are generally distinct from the bulk of the emVE, however one cannot rule out temporal dynamics in gene expression and/or perdurance of any of these reporters giving rise to heterogeneity. The interrelationship of the AVE and emVE populations is not well understood, and questions, such as whether they are distinct as far back as the PrE of the blastocyst, will need to be established using other approaches, for example single-cell-level transcriptomic analyses. The emVE will go on to form the future gut endoderm together with DE cells which arise from the pluripotent epiblast at gastrulation, and which also express *Hex* (Rodriguez et al., 2001), and which were positive for Tomato expression. Additionally, the midline and axial mesendoderm cells expressed the *Hex-tdTomato*, but not the *Afp-GFP*, reporter (Fig. 6E-H).

Due to their overlapping and/or exclusive expression patterns, dual reporter combinations such as the *Hex-tdTomato*; *Pdgfra^H2BGFP/+^* and *Hex-tdTomato*; *Afp-GFP* will be valuable tools for future studies investigating the behavior of neighboring cells during the DVE/AVE migration process, as well as during gut endoderm, midline and notochord formation. These dual-labeled transgenics also reveal an inherent heterogeneity within the endoderm and can be used to select distinct subsets of cells for transcriptomic analyses with the goal of understanding the molecular divergence and relation of various endoderm lineages.

### Visualizing cellular behaviors during the collective cell migration of the AVE

**Fig. 7.**
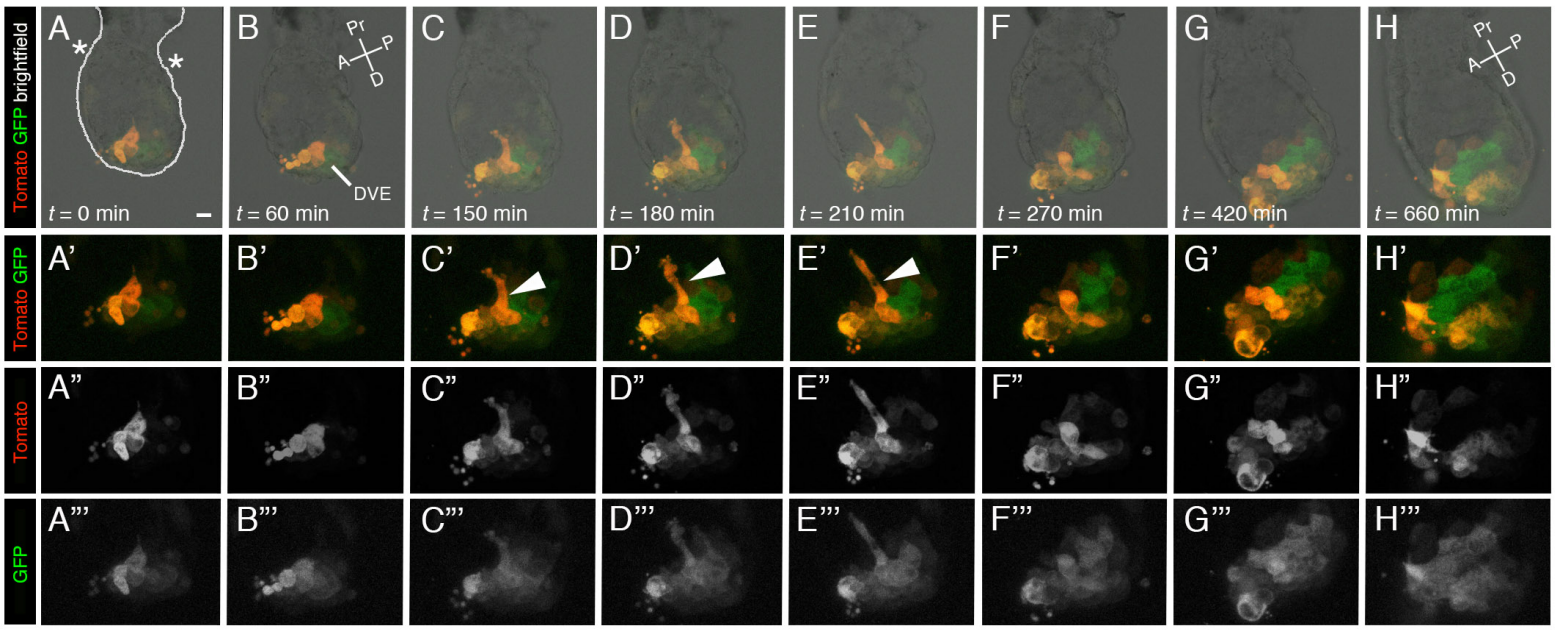
**Live imaging a *Hex-tdTomato*; *Hex-GFP* double transgenic dual reporter embryo reveals cellular behaviors associated with migration of DVE/AVE cells.** (A-H) Representative time frames taken from a 3D time lapse imaging experiment of a E5.5 *Hex-tdTomato; Hex-GFP* over a period of 660 min. The embryo was imaged every 10 min. The series depicts the initiation of AVE migration. Tomato- and GFP-positive cells can be seen to migrate towards the future anterior of the embryo. (A-H) Panels show merge of red and green channels with bright field image of whole mount view of embryo. (A) White line outlines the contour of the embryo. Note, white arrows point to anteriorly oriented protrusions of AVE cells. (A′-H‴) Zoomed in views of the distal part of the embryo, corresponding to the region of the VE overlying the epiblast. (A′-H′) Tomato and GFP fluorescence. (A″-H″) Tomato fluorescence. (A‴-H‴) GFP fluorescence. Asterisks mark the border of extra-embryonic and embryonic visceral endoderm. A, anterior; D, distal; DVE, distal visceral endoderm; P, posterior; Pr, proximal; Scale bar: 20 µm.

To evaluate whether the *Hex-tdTomato* is suitable for dual-color live imaging we generated *Hex-tdTomato*; *Hex-GFP* double transgenic embryos and 3D time-lapse imaged the start of AVE migration (Fig. 7). We dissected litters from *Hex-tdTomato*; *Hex-GFP* dual reporter matings at day E5.5. Both reporters were readily detected and localized to a population of cells located at the distal tip (the DVE) of recovered double transgenic embryos, at the start of the time-lapse. Expression of *Hex-tdTomato* appeared slightly brighter than *Hex-GFP* throughout the time-lapse. The fluorescence intensity of each reporter at this stage was sufficient for live imaging of embryos for a prolonged period of time; however, due to its increased fluorescence intensity, the *Hex-tdTomato* may present a more desirable reporter for increased time or z resolution imaging, or decreased time of acquisition at these early stages. Over the time course of the experiment the number of cells labeled by the reporters and comprising the AVE expanded as the population migrated unilaterally towards the future anterior of the embryo. During this collective cell migration, a subset of cells were seen to extend anteriorward protrusions which projected toward the extra-embryonic region (Fig. 7C-E, white arrows in 7C′-E′, 7E″-E″′) (Omelchenko et al., 2014; Srinivas et al., 2004). These protrusions were highly dynamic, extensively remodeled, and only present on cells for about an hour. Some cells showed elevated levels of fluorescence, including those cells extending the protrusions which were generally located at the front of the migrating population. Indeed, AVE cells exhibiting extended protrusions have been proposed to initiate the collective migration associated with the DVE/AVE population (Morris et al., 2012).

It has been over a decade since the first studies using the *Hex-GFP* reporter to live image the cellular behaviors associated with DVE/AVE migration (Rodriguez et al., 2005; Srinivas et al., 2004), and even after numerous studies, several open questions remain. The high proliferation rate of the underlying epiblast could influence the reorganization of the overlying VE epithelium, including the DVE/AVE. Furthermore, cell shape changes throughout the VE could impact the trans-epithelial movement of DVE/AVE. Exploiting two spectrally-distinct reporters to live image the DVE/AVE in relation to its neighbors, for example the entire VE cell population which we have shown can be labeled with *Afp* or *Transthyretin* (*Ttr*; Kwon and Hadjantonakis, 2009) *cis*-regulatory element based transgenics, should provide the necessary resolution to address some of these questions.

To date, a small cohort of genetically encoded fluorescent protein reporter mouse lines suitable for imaging the DVE/AVE exist, including *Hex-GFP*, *Cerl-GFP* and *Lefty-mVenus* (Mesnard et al., 2004; Rodriguez et al., 2001; Takaoka et al., 2011). A recent study has reported three nuclear-localized H2B-mCherry-based reporters; *Cerl-H2B-mCherry*, *Dkk1-H2B-mCherry*, *Lefty1-H2B-mCherry*, *Otx2-H2B-mCherry* (Hoshino et al., 2015). Since the available cytoplasmic reporters which delineate details of cellular morphology are based on GFP, or its variant Venus, they cannot readily be combined with one another nor with the majority of available reporters that label neighboring cell populations, such as the pan VE *Afp-GFP* reporter (Kwon et al., 2006), or early panVE and later mesoderm-specific reporter *Pdgfra^H2BGFP^* (Artus et al., 2010) used in this study. Consequently a spectrally-distinct cytoplasmic RFP-based *Hex-tdTomato* reporter that can reveal cellular morphology and be readily be distinguished from the expanding cohort GFP-based reporters represents a requisite tool facilitating the combinatorial multi-spectral imaging that will be required to address key open questions concerning the complex cellular dynamics of the endoderm, which undoubtedly involve interactions between the neighboring populations of cells.

## MATERIAL AND METHODS

### Transgenic construct generation and mouse strain establishment

The *Hex-tdTomato* construct design was based on the previously published *Hex-GFP* plasmid vector (Rodriguez et al., 2001). *Pme*I and *Pac*I restriction sites were introduced to the *Hex-GFP* plasmid vector before and after the GFP coding sequence, respectively. The coding sequence for *tdTomato* was PCR amplified from p*RSET-BtdTomato* (Shaner et al., 2004) using the following primers: tdTomato-For:

5′-AATCCTAGGATTGATATCGTTTAAACGCCACCATGGTGAGCAAGGGCGAGGA’ and tdTomato-Rev: 5′-AATATTTAAATCCGTTAATTAAATCGATTCGCGATTACTTGTACAGCTCGTCCAT-3′.

The resulting PCR product was cloned into the original *Hex-GFP* plasmid using the introduced *Pme*I and *Pac*I sites to excise and replace the *GFP* with *tdTomato*. The *Hex-tdTomato* construct was confirmed by DNA sequencing.

To generate the *Hex-tdTomato* transgenic mouse line, *Hex-tdTomato* plasmid DNA was linearized with *Kpn*I, subject to agarose gel electophoresis, purified using the Qiagen Gel Purification kit, and microinjected into the pronucleus of C57BL/B6 zygotes by the Memorial Sloan-Kettering Cancer Center Transgenic Core Facility (New York, NY, USA). Positive founder (F0) animals were identified by PCR using the following primers: tdTomato For-1: 5′-CTACCTGGTGGAGTTCAAGA-3′ and tdTomato Rev-1: 5′-GCCATGTTGTTGTCCTCGGA-3′; and tdTomato For-2: 5′-GCGAGGAGGTCATCAAAGAG-3′ and tdTomato Rev-2: 5′-CCCATGGTCTTCTTCTGCAT -3′. The sizes of amplicons of each set primers are 228 bp and 415 bp, respectively.

### Cell culture and transfection

Caco2 (ATCC HTB-37) and Hepa1-6 (ATCC CRL1830) cells were cultured in DMEM containing GlutaMax (Invitrogen) supplemented with 10% fetal calf serum (FCS), penicillin, and streptomycin (Gibco; Life Technologies). Cells were transfected with Lipofectamine 2000 (Invitrogen) according to manufacturer's recommendations and imaged for fluorescence within 24–48 h.

### Mouse husbandry

The experimental procedures were approved by the Memorial Sloan Kettering Cancer Center's Institutional Animal Care and Use Committee. All procedures involving experimental animals were performed in compliance with local animal welfare laws, guidelines and policies. Three male and two female founders (F0 animals) were identified as positive by PCR from a total of forty animals recovered after pronuclear injection of zygotes. These five founder animals were crossed to CD1 wild-type females and males respectively, with transgenic offspring (F1 animals) identified by PCR, and screened for robust endoderm localized red fluorescence in post-implantation embryos at different developmental stages. Of these five founders, two (#7, #41) exhibited robust, uniform and comparable expression of tdTomato. Embryos from F1 animals of male founder #7 were analyzed in detail and are presented in this report. All data presented are of hemizygous (tg/+) transgenic *Hex-tdTomato* animals. To study the co-localization of *Hex-tdTomato* with *Afp-GFP*, *Hex-GFP* and *Pdgfra^H2BGFP^* green fluorescent reporters, F1 *Hex-tdTomato* mice were crossed to either *Afp-GFP* (Kwon et al., 2006), *Hex-GFP* (Rodriguez et al., 2001) or *Pdgfra^H2BGFP/+^* (Hamilton et al., 2003) mouse lines to generate double transgenic dual color reporter expressing embryos.

### Embryo dissection and sectioning

E0.5 was defined as noon of the day when a vaginal plug was detected in pregnant females. Embryos at the indicated stages were dissected in DMEM-F12 media containing 5% newborn calf serum. Embryos were fixed in 4% paraformaldehyde in phosphate-buffered saline (PBS) for 20 min, and then washed three times in 0.1% PBT (PBS with 0.1% Triton X-100). Fixed embryos were counterstaining with Hoechst 33342 (1:500; Invitrogen) to label nuclei and Phalloidin (1:1000) to label F-Actin. tdTomato and GFP were visualized directly in all preparations (live and fixed tissue) presented in this study.

For cryosections, embryos were equilibrated in 10% sucrose in PBS, then in 30% sucrose in PBS for 3 h and OCT (Tissue-Tek, SAKURA FINETEK USA INC.) overnight at 4°C. They were then snap-frozen in OCT. Sections were cut with a Leica cryostat at 12 μm. Sections were mounted onto glass slides in Fluoromount-G (Southern Biotech, Birmingham, AL, USA) and imaged through glass coverslips.

### Embryo culture and time-lapse imaging

Embryos were dissected in DMEM/F12 containing 5% newborn calf serum, and then cultured in medium comprising 50% DMEM/F12 Glutamax/50% Rat Serum as previously described (Nowotschin et al., 2010). The embryo depicted in Fig. 7 was imaged for a total of 11 h in culture medium on the microscope stage at 10 min time intervals and 2.29 µm z intervals.

### Image data acquisition and processing

E9.5 embryos were imaged on a Leica M165FC stereo-dissecting microscope using a Zeiss Axiocam MRm CCD camera. Raw data were processed using Axiovision/ZEN Blue software. Laser scanning confocal data were acquired on a Zeiss LSM880 laser scanning confocal microscope. Fluorophores were excited using a 405-nm diode laser (Hoechst), 488-nm argon laser (GFP), DPSS-561-10-nm laser (tdTomato) or 633 nm HeNe (far red). Objectives used were a Plan-Apo 20×/NA0.75 and Plan-Apo 40×/1.4 Oil DIC M27. Embryos were imaged in wholemount in MatTek dishes (Ashland, MA, USA). Confocal images were acquired as *z*-stacks of x–y images taken at 0.5–2 μm *z* intervals. Raw confocal data were processed using ZEN software (Carl Zeiss Microsystems, http://www.zeiss.com) and Imaris 8.2 software (Bitplane, www.bitplane.com). Images were post-processed in CS6 Photoshop (Adobe).
